# Clinical features of 2041 human brucellosis cases in China

**DOI:** 10.1371/journal.pone.0205500

**Published:** 2018-11-26

**Authors:** Yujing Shi, Hui Gao, Georgios Pappas, Qiulan Chen, Mei Li, Jun Xu, Shengjie Lai, Qiaohong Liao, Wenwen Yang, Zhongtao Yi, Zulaguli Rouzi, Hongjie Yu

**Affiliations:** 1 Division of Infectious Diseases, Key Laboratory of Surveillance and Early–warning on Infectious Disease, Chinese Center for Disease Control and Prevention, Beijing, China; 2 The Sixth People's Hospital of the Xinjiang Uygur Autonomous Region, Urumqi, China; 3 University Hospital of Ioannina, Ioannina, Greece; 4 School of Public Health, Fudan University, Key Laboratory of Public Health Safety, Ministry of Education, Shanghai, China; 5 Hubei Provincial Center for Disease Control and Prevention, Wuhan, China; 6 Hubei Provincial Center for Disease Control and Prevention, Wuhan, China; Aga Khan University - Kenya, KENYA

## Abstract

**Background:**

Human brucellosis has become a major public health problem in China. However, the available clinical data on brucellosis cases are limited.

**Methods:**

We retrospectively reviewed medical charts of 2041 patients with confirmed human brucellosis and prospectively recorded their outcomes by telephone interview. These patients were admitted to the Sixth People’s Hospital of the Xinjiang Uygur Autonomous region between 1^st^ January and 31^st^ December 2014. Data on these patients were collected from hospital medical records.

**Results:**

Many patients presented with fatigue (67%), fever (64%), arthralgia (63%) and sweating (54%). High erythrocyte sedimentation rate (ESR) (69%), high C-reactive protein (CRP) (39%), high alanine aminotransferase (ALT) (33%) and high aspartate aminotransferase (AST) (20%) were the most common laboratory findings, especially in acute patients. There was evidence of focal involvement in 90% of patients. A total of 61.5% of brucellosis patients recovered. Multivariate logistic regression analyses suggested that the risk factors key to unfavorable prognosis were: age≥45 years (OR = 1.75, 95% CI 1.36–2.24), back pain (OR = 1.50, 95% CI 1.16–1.94) and joint tenderness (OR = 1.73, 95% CI 1.13–2.65). The increasing duration of the illness increased the risk of poor prognosis.

**Conclusion:**

Patients with brucellosis showed different characteristics in different clinical stages. In China, the chronicity rate of human brucellosis is high. The risk of poor prognosis is increased in patients aged 45 years or older, patients who have had brucellosis for a substantial period of time, and patients with back pain or joint tenderness. The clinical management of brucellosis should be improved to include sensitive diagnostic methods for subacute and chronic brucellosis.

## Introduction

Brucellosis, caused by bacteria of the genus Brucella, is a major zoonosis worldwide. Although it has been well controlled in most developed countries, brucellosis is still endemic in Africa, Asia, the Middle East, the Mediterranean and South America [1, 2]. Human brucellosis is a major public health problem that has re-emerged in China since the mid-1990s, with the highest recorded number of cases (56,989 cases) in 2015 [3–5]. This may be due to the dynamic growth of animal husbandry in China, which enhances the chance of human infection. Moreover, the national infectious disease surveillance system has reported an increased rate of human brucellosis [6].

The disease is spread to humans mainly by the ingestion of infected meat or unpasteurized dairy products, by contact with infected animals or inhalation of infectious aerosolized particles [7]. Brucellosis usually leads to protean manifestation and may have polymorphic features affecting any organ system. The diagnosis of brucellosis is challenging because unusual presentations and non-specific symptoms can lead to misdiagnosis and treatment delay. Although the mortality rate is low, brucellosis can be severely debilitating and disabling [8, 9]. A timely and accurate diagnosis is key to the clinical management of brucellosis. There is an urgent need for an increased awareness of the clinical characteristics of brucellosis among physicians.

Between 2000 and 2012, research into the clinical characteristics of brucellosis was done in Turkey [10], Iran [11], Greece [12] and Saudi Arabia [13]. But the results differed between studies. This may be due to small sample sizes or different methods for case classification. In order to reliably examine the clinical characteristics of brucellosis, the series used in the present study is larger than that used in previous studies, and a common case classification is used.

Reliable data on the clinical characteristics of human brucellosis should improve the identification of relevant prognostic factors and improve the clinical management of this debilitating condition. This study aims to describe the symptoms, signs and laboratory findings of 2041 hospitalized patients with laboratory confirmed brucellosis, and assess the risk factors for their clinical outcomes.

## Methods

### Setting

The Sixth People's Hospital of the Xinjiang Uygur Autonomous Region (hereafter called the Sixth People’s Hospital) is a tertiary infectious disease specialist hospital with 11 clinical departments and 460 beds. As an important referral center, the hospital approximately reported 27% of total reported brucellosis cases in Xinjiang yearly. A total of 2041 patients with laboratory-confirmed brucellosis were admitted to the hospital between January 1^st^ and December 31^st^ of 2014 and were included in this retrospective study. We accessed patient medical records from March 12^th^ to early April 2016 and performed telephone interviews in late April 2016. The patient files were examined using a standardized form, which recorded demographic data, medical history, clinical and laboratory findings, any antibiotic treatment, and any focal involvements.

### Case definition

A confirmed case of brucellosis was defined as a patient with compatible clinical symptoms (such as arthralgia, fever, sweating, chills, headache, myalgia, malaise) and laboratory evidence of Brucella infection diagnosed by bacteria culture or SAT (≥1:200). Based on the duration of the systemic disease before admission to hospital, patients were divided into three groups: acute brucellosis (<2 months), subacute brucellosis (2–12 months), and chronic brucellosis (>12 months). All cases underwent routine laboratory tests.

### Treatment and follow-up

Patients were treated with various combinations of antibiotics. The antibiotics regimens were given on the basis of China’s Practice Guideline for brucellosis diagnosis and treatment [14]: doxycycline (100mg every 12 h), rifampin (600 -900mg every 24 h), intramuscular streptomycin (15mg/kg every 24 h), levofloxacin (200mg every 12 h), ciprofloxacin (750mg every 12 h), and co-trimoxazole (960mg every 12 h). For children under 8 years of age and pregnant women, only rifampin (600-900mg every 24 h) was provided. Given the effectiveness of previous treatment and patient compliance (Xinjiang’s vast land posed a challenge to subsequent patient visits), the treatment duration was extended to 3–6 months for all patients. The outcome of treatment (including medication compliance and symptom recovery) was investigated by telephone interview. Admittedly telephone interview is not the ideal method of follow-up, but since in chronic/ persisting brucellosis the symptoms are often subjective and telephone interview allowed for a larger enrollment of patients in follow-up, we considered this to be an acceptable follow-up method.

### Data analysis

Data were entered into Epidata (version 2.0, Odense, Denmark). Medians and interquartile ranges (IQRs) were calculated for continuous variables, and compared between different groups using the Wilcoxon rank sum test. For categorical variables, case frequencies in each category were compared using a Chi-square test or Fisher’s exact test. For multiple comparisons, the Bonferroni correction was applied. To further examine the association between potential risk factors and treatment outcome, we first performed univariate analysis. For multivariable logistic regression, we included variables with *p<*0.05 in univariate analysis or those believed to be potential risk factors associated with the outcome. The software program SAS 9.3 and R 3.3.1 were used to analyze the data.

### Ethical approval

Ethical approval (no. 201533) was obtained from the Institutional Review Board (IRB) of China CDC before the survey began. With IRB’s ratification, we signed a confidentiality agreement with the hospital to use patient medical records for research purposes. We confirmed that all participant identifying information (including patient names, ID numbers, home addresses and telephone numbers) would not be included in recordings, written descriptions or publications. Because written informed consent is difficult to obtain during a telephone survey, the use of verbal informed consent was approved by the IRB for this retrospective study. Verbal informed consent was obtained from all respondents over the phone and documented in forms. For subjects younger than 17 years of age, we obtained verbal consent from their parents or legal guardians.

## Results

**Fig 1:**
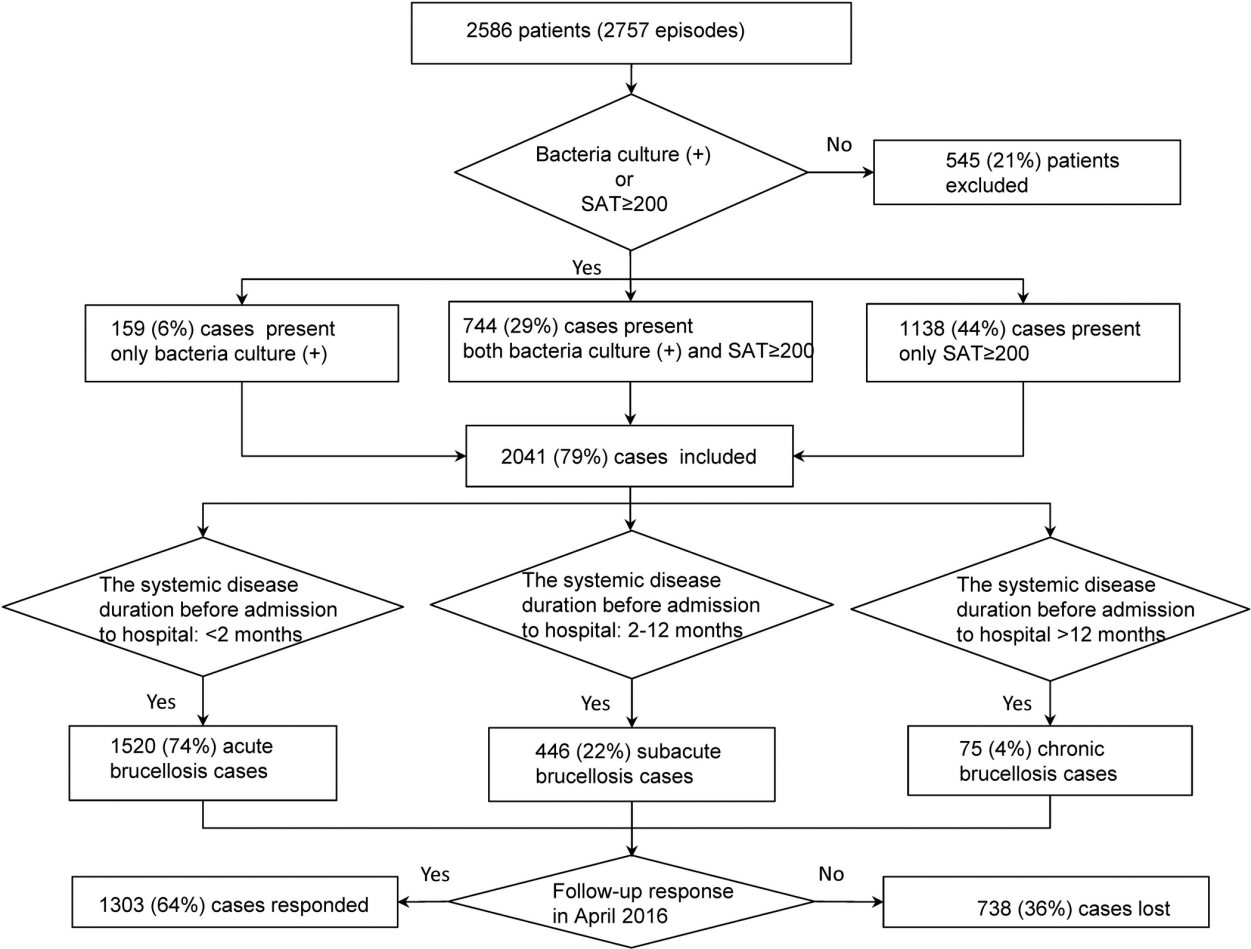
Flowchart for enrollment of 2041 brucellosis confirmed cases in Xinjiang, China, 2014.

A total of 2586 patient files (including 2757 hospitalization records) were investigated for the purpose of this study. For patients who were hospitalized more than once between January 1^st^ and December 31^st^ of 2014, only their first hospitalization records were evaluated. A total of 2041 laboratory confirmed cases met the case definition and inclusion criteria and were included in this study (see Fig 1). The median age of the 2041 cases was 43 years (IQR 21–52) and 77% were male. A total of 1141 patients (56%) were aged between 25–49 years and 529 (26%) were aged between 50–64 years. The age distribution was significantly different between acute, subacute and chronic patients, *p =* 0.001. Most (87%) patients under 15 years-old presented as acute cases. Of 2041 cases, 90% were from rural areas, 84% were farmers or herders, 97% had a history of animal exposure and 1.8% ingested unpasteurized foods. For patients whose first contact health sector was the Sixth People’s Hospital, the median number of days from illness onset to diagnosis was 12 days (IQR 7–28) in acute cases, 92 days (IQR 68–129) in subacute cases and 410 days (IQR 369–784) in chronic cases (Table 1).

**Table 1:** Demographic features and exposure history of 2041 brucellosis cases, Xinjiang, China, 2014.

Characteristics	Acute (N = 1520)	Subacute (N = 446)	Chronic (N = 75)	Total (N = 2041)	p*
Demographic features					
Male sex	1180 (78)	338 (76)	55 (73)	1573 (77)	0.53
Age group (years)					
<4	36 (2)	5 (1)	-	41 (2)	0.001
5–14	63 (4)	10 (2)	-	73 (4)	
15–24	115 (8)	32 (7)	2 (3)	149 (7)	
25–49	871 (57)	229 (51)	41 (55)	1141 (56)	
50–64	362 (24)	139 (31)	28 (37)	529 (26)	
≥65	72 (5)	32 (7)	4 (5)	108 (5)	
From					
Urban	150 (10)	52 (12)	8 (11)	210 (10)	0.55
Rural	1370 (90)	394 (88)	67 (89)	1831 (90)	
Occupation					
Farmer& herdman	1276 (84)	373 (84)	64 (85)	1713 (84)	0.30
Student	67 (4.4)	16 (3.6)	-	83 (4.1)	
Preschool children	46 (3.0)	8 (1.8)	-	54 (2.7)	
Veterinarian	37 (2.4)	9 (2.0)	5 (6.7)	51 (2.5)	
Other occupation	244 (16)	73 (16)	11 (15)	328 (16)	
Exposure history					
Animals exposure	1479 (97)	431(97)	71 (96)	1981 (97)	0.52
Sheep & goats	1465 (96)	427 (96)	71 (95)	1963 (96)	0.92
Cattle	1356 (89)	382 (86)	63 (84)	1801 (88)	0.30
Other animals*	11 (0.7)	6 (1)	1 (1)	18 (0.9)	0.08
Unknown	42 (3)	14 (3)	3 (4)	60 (3)	0.77
Method of exposures to animals					
Raising	1401 (92)	401 (90)	63 (84)	1865 (91)	0.08
Delivering lambs	934 (61)	298 (67)	56 (75)	1288 (63)	0.04
Slaughter	410 (27)	145 (33)	26 (35)	581 (28)	0.14
Others routes**	70 (5)	25 (6)	13 (17)	108 (5)	<0.0001
Ingestion of unpasteurized food	23 (1.5)	13 (3)	1 (1)	37 (1.8)	0.20
Days from onset to diagnosis^†^	12 (7–28)	92 (68–129)	410(369–784)	15 (7–33)	-
Duration of hospitalization (days)	12 (9–14)	13 (10–15)	13 (10–15)	12 (9–14)	0.07

Data are no. (%) of case, unless otherwise indicated. Percentages may not total 100 because of rounding. IQR, inter quartile range.

†Only 432 acute cases, 65 subacute cases and 9 chronic cases whose first contact health sector was the Sixth People’s Hospital were enrolled for analysis. Patients who were referred to the current hospital and diagnosed at other hospitals were not included.

*Other animals include pigs, dogs, horses, deer and camels.

**Other routes include veterinarian, vaccine producing, animal trade, animal product processing and sheep clipping.

### Clinical presentation

Table 2 lists the main symptoms and signs on presentation. Based on the systemic disease duration before admission to hospital, 1520 (74%) cases were evaluated as acute, 446 (22%) cases as subacute and 75 (4%) as chronic (Fig 1). The most common symptoms on presentation were fatigue (67%), arthralgia (63%), sweating (54%), back pain (37%) and lack of appetite (25%). The most common signs were fever (64%), splenomegaly (42%), hepatomegaly (24%) and weight loss (19%). In a comparison of different clinical groups, fever, splenomegaly, lack of appetite and cough were more common in acute cases (68%, 45%, 27%, 12%, respectively) than in subacute (56%, 36%, 19%, 5%, respectively, adjusted *p*<0.05) and chronic cases (43%, 21%, 15%, 3%, respectively, adjusted *p*<0.05). A significantly lower number of acute cases presented with arthralgia, back pain and limited motion (60%, 32%, 18%, respectively) than subacute (70%, 49%, 26%, respectively, adjusted *p*<0.05) and chronic cases (79%, 53%, 39%, respectively, adjusted *p*<0.05). There were significant adjusted differences in fatigue between subacute and chronic cases (adjusted *p* = 0.02), and differences in weight loss between acute and chronic cases (adjusted *p* = 0.03).

**Table 2:** Symptoms and signs of 2041 brucellosis cases according to clinical stage, Xinjiang, China, 2014.

	Acute (N = 1520)	Subacute (N = 446)	Chronic (N = 75)	Total (N = 2041)	*p* *	Comparisons among groups *(Bonferroni correction)*
Symptoms						
Fatigue	1002 (66)	320 (72)	42 (56)	1364 (67)	0.01	Subacute > Chronic
Arthralgia	911 (60)	313 (70)	59 (79)	1283 (63)	<0.0001	Acute < Subacute, Chronic
Sweating	824 (54)	242 (54)	39 (52)	1105 (54)	0.93	n.s
Back pain	491 (32)	219 (49)	40 (53)	750 (37)	<0.0001	Acute < Subacute, Chronic
Lack of appetite	407 (27)	84 (19)	11 (15)	502 (25)	0.0004	Acute > Subacute, Chronic
Headache	381 (25)	104 (23)	15 (20)	500 (24)	0.48	n.s
Limited motion	271 (18)	115 (26)	29 (39)	415 (20)	<0. 0001	Acute < Subacute, Chronic
Chills	237 (16)	61 (14)	12 (16)	310 (15)	0.59	n.s
Myalgia	189 (12)	49 (11)	8 (11)	246 (12)	0.65	n.s
Sleep disturbance	165 (11)	55 (12)	4 (5)	224 (11)	0.19	n.s
Cough	175 (12)	21 (5)	2 (3)	198 (10)	<0.0001	Acute > Subacute, Chronic
Nausea	82 (5)	21 (5)	7 (9)	110 (5)	0.24	n.s
Orchialgia	69 (5)	11 (2)	1 (1)	81 (4)	0.08	n.s
Vomitting	36 (2)	11 (2)	1 (1)	48 (2)	0.96	n.s
Abdominal pain	17 (1.1)	7 (1.6)	4 (5.3)	28 (1.4)	0.02	n.s
Signs						
Fever^†^	1031 (68)	251 (56)	32 (43)	1314 (64)	<0.0001	Acute > Subacute, Chronic
Splenomegaly	678 (45)	159 (36)	16 (21)	853 (42)	<0.0001	Acute > Subacute>Chronic
Hepatomegaly	362 (24)	106 (24)	13 (17)	481 (24)	0.43	n.s
Weight loss	313 (21)	76 (17)	7 (9)	396 (19)	0.02	Acute > Chronic
Joints tenderness	121 (8)	45(10)	12 (16)	178 (9)	0.03	n.s
Testis swelling	49 (3)	7 (2)	1 (1)	57 (3)	0.15	n.s
Joints swelling	29 (2)	14 (3)	5 (7)	48 (2)	0.02	n.s
Lymphadenopathy	25 (2)	8 (2)	3 (4)	36 (2)	0.25	n.s
Others¶	19 (1)	2 (0.4)	1 (1)	22 (1)	0.03	n.s

Data are no. (%) of cases, unless otherwise indicated. Percentages may not total 100 because of rounding.

†Fever was defined as axillary and rectal temperature of >37.3°Cand >38.3°C, respectively.

¶Include rash (12), joints deformity (4), meningeal irritation (2) and jaundice (1) in acute brucellosis, rash (2) in subacute brucellosis and cardiac murmur (1) in chronic brucellosis.

*Percentages of cases with each type were compared with a Chi-square test or Fisher’s exact test (where 20% cells have expected count less than 5).

n.s: adjusted *p*≥0.05, not significant

### Laboratory findings

Laboratory findings at admission are shown in Table 3. The most common laboratory findings were high ESR (69%), high CRP (39%), high ALT (33%) and high AST (20%). These abnormal findings more frequently occurred in acute cases than in the other two groups, adjusted *p<*0.05 (S1 Fig). Anemia was more commonly seen in acute cases (26%) than in subacute cases (20%), adjusted *p*<0.05. Across all patients, the median hemoglobin was 129 g/L (IQR 117–139), median leukocyte count was 5.9×10^9^ cells/L (IQR 4.7–7.1), median lymphocyte count was 2.8×10^9^ cells/L (IQR 2.1–3.7) and median thrombocyte count was 221×10^9^ /L (IQR 175–272). The median CRP and ESR in the total group were 6.6 mg/L (IQR 2.4–20.0) and 28 mm/h (15–46) respectively. A total of 903 of 1925 cases (47%) showed Brucella growth in their blood culture. And the positive rate (53%) in acute cases was significantly higher than in subacute (31%) and chronic groups (19%) (adjusted *p<*0.0001). The positive rate of SAT of acute and subacute cases gradually decreased along with the progress of the condition after treatment (S2 Fig). The results of regular re-examinations after discharge showed that most acute patients laboratory tests improved after treatment (S3 Table).

**Table 3:** Laboratory findings of 2041 brucellosis cases according to clinical stage, Xinjiang, China, 2014.

Variables	Acute (n = 1520)	Subacute (n = 446)	Chronic (n = 75)	Total (n = 2041)	p*	Comparisons among groups *(Bonferroni correction)*
Haematology						
Anemia†	387 (26)	87 (20)	14 (19)	488 (24)	0.01	Acute > Subacute
Median Hb (IQR) (g/L)	128 (116–139)	129 (119–140)	132 (121–142)	129 (117–139)	0.006	Acute < Chronic
Leukopenia, <4×10^9^/L	190 (12)	43 (10)	8 (11)	241 (12)	0.24	n.s
Leukocytosis, >10×10^9^/L	91 (6)	18 (4)	4 (5)	113 (6)	0.29	n.s
Median WBC count(IQR)	5.9 (4.7–7.2)	5.8 (4.7–7.0)	5.9 (4.7–7.1)	5.9 (4.7–7.1)	0.82	n.s
Lymphopenia, <0.8×10^9^/L	9 (0.6)	2 (0.5)	0	11 (0.5)	0.76	n.s
Lymphocytosis, >4×10^9^/L	291 (19)	81 (18)	18 (24)	390 (19)	0.49	n.s
Median LYM count(IQR)	2.8 (2.1–3.7)	2.8 (2.0–3.7)	3.0 (2.3–4.0)	2.8 (2.1–3.7)	0.30	n.s
Thrombocytopenia,<100×10^9^/L	57 (4)	19 (4)	3 (4)	79 (4)	0.88	n.s
Median PLT count(IQR)	221(175–273)	220 (173–269)	225 (193–267)	221 (175–272)	0.42	n.s
Serum biochemistry						
ALT>40 U/L	568 (37)	99 (22)	8 (11)	675 (33)	<0.0001	Acute > Subacute> Chronic
Median ALT (IQR)	36 (23–60)	25 (17–43)	20 (14–30)	32 (21–54)	<0.0001	Acute > Subacute> Chronic
AST>42 U/L	355 (23)	56 (12)	3 (4)	414 (20)	<0.0001	Acute > Subacute> Chronic
Median AST (IQR)	28 (20–44)	22 (16–30)	18 (14–25)	26 (18–40)	<0.0001	Acute > Subacute> Chronic
Bilirubin >18.6 umol /L	225 (15)	48 (11)	6 (8)	279 (14)	0.03	n.s
Median bilirubin (IQR)	10.3 (7.2–15.2)	9.9 (7.0–13.4)	9.5 (6.2–13.0)	10.1 (7.1–14.6)	0.01	n.s
Urea nitrogen >7.14 mmol/L	48 (3)	20 (4)	6 (8)	74 (4)	0.05	n.s
Median urea nitrogen (IQR)	4.1 (3.4–5.0)	4.4 (3.5–5.3)	4.5 (3.7–5.1)	4.2 (3.4–5.1)	0.0002	Acute < Subacute
Creatinine >124 umol/L	8 (0.5)	3 (0.7)	0	11 (0.5)	0.80	n.s
Median creatinine (IQR)	60 (51–68)	58 (50–66)	59 (51–68)	59 (51–68)	0.03	Acute > Subacute
Inflammatory markers						
CRP >10 mg/L	643 /1515(42)	143 (32)	17 (23)	803 (39)	<0.0001	Acute > Subacute, Chronic
Median CRP(IQR)	7.6 (3.0–21.9)	4.2 (1.3–14.7)	2.3 (0.9–7.6)	6.6 (2.4–20.0)	<0.0001	Acute > Subacute, Chronic
ESR elevation¶	1088 (72)	280 (63)	43 (57)	1411 (69)	0.0002	Acute > Subacute, Chronic
Median ESR (IQR)	29 (16–46)	26 (12–47)	22 (12–40)	28 (15–46)	0.0015	Acute > Subacute, Chronic
Bacterial culture and serum-antibody-test						
Culture positive	758/1426 (53)	131/424 (31)	14/75 (19)	903/1925(47)	<0.0001	Acute > Subacute, Chronic
SAT ≥200	1394 (92)	419 (94)	69(92)	1882 (92)	0.33	n.s
SAT ≥400	996 (66)	261 (59)	38 (51)	1295 (63)	0.002	Acute > Subacute, Chronic
Median SAT titer(IQR)	400 (200–400)	400 (200–400)	400 (200–400)	400 (200–400)	0.007	Subacute > Chronic

Data are no. (%) of cases, unless otherwise indicated. Percentages may not total 100 because of rounding. Abbreviation: ALT, alanine aminotransferase; AST, aspartate aminotransferase; ESR, erythrocyte sedimentation rate; CRP, C-reactive protein; SAT, standard tube agglutination test;

† Anemia: female and children <110 g/L, male<120 g/ L.

¶ESR positive: female>20 mm/h and male>15 mm/h.

*Medians were compared between each group with the Wilcoxon rank sum test. For categorical variables, percentages of cases in each group were compared with Chi-square test or Fisher’s exact test (where 20% cells have expected count less than 5).

n.s: adjusted *p*≥0.05, not significant

### Focal involvement

There was evidence of focal involvement in 1829 cases (90%) (see Table 4). Osteoarticular involvement was the most frequent and occurred in 1380 cases (68%), including peripheral arthritis (57%), spondylitis (20%) and sacroiliitis (2%). Osteoarticular involvement was found to be significantly more frequent in chronic (87%) and subacute (77%) cases compared to acute cases (64%) (adjusted *p<*0.001).

**Table 4:** Focal involvements of 2041 brucellosis cases during clinical stage, Xinjiang, China, 2014.

Focal Involvement†	Acute (n = 1520)	Subacute (N = 446)	Chronic (N = 75)	Total (N = 2041)	p*	Comparisons among groups *(Bonferroni correction)*
Osteoarticular	970 (64)	345 (77)	65(87)	1380 (68)	<0.0001	Acute < Subacute, Chronic
Peripheral arthritis	823 (54)	288 (64)	56 (75)	1167 (57)	<0.0001	Acute < Subacute, Chronic
Spondylitis	255 (17)	119 (27)	25 (33)	399 (20)	<0.0001	Acute < Subacute, Chronic
Sacroiliitis	36 (2)	12 (3)	0	48 (2)	0.45	n.s
Gastrointestinal	804 (53)	213 (48)	25(33)	1042 (51)	0.001	Acute, Subacute >Chronic
Haematological∮	547 (36)	129 (28)	20 (27)	696 (34)	0.0084	Acute>Subacute
Genitourinary	296 (19)	100 (22)	13 (17)	409 (20)	0.34	n.s
Epididymo-orchitis	273 /1180(23)	95/338 (28)	13/55 (24)	381/1573 (24)	0.17	n.s
PID§	23/340 (7)	5 /108(5)	0	28 /468(6)	0.11	n.s
Respiratory	112 (7)	10 (2)	0	122 (6)	<0.0001	Acute > Subacute, Chronic
Bronchitis	90 (6)	2 (0.4)	0	92 (5)	<0.0001	Acute > Subacute, Chronic
Pneumonia	15 (1)	1(0.2)	0	16 (0.8)	0.26	n.s
Pleural adhesions	10 (0.7)	6 (1)	0	16 (0.8)	0.31	n.s
Cardiovascular#	8 (0.5)	5 (1)	0	13 (0.6)	0.34	n.s
Cutaneous	12 (0.8)	2 (0.5)	0	14 (0.7)	0.13	n.s
CNS¶	4 (0.3)	0	1(1)	5 (0.3)	0.63	n.s
Uveitis	0	1 (0.2)	0	1 (0.05)	0.25	n.s

Data are no. (%) of cases, unless otherwise indicated. Percentages may not total 100 because of rounding.

† Focal involvements not mutually exclusive; some patients had multiple focal involvements.

∮Includes anemia, leukopenia, leukocytosis, lymphopenia, lymphocytosis and thrombocytopenia.

§PID: Pelvic Inflammatory Disease

# Includes endocarditis (2) and pericarditis (11).

¶ CNS = central nervous system, includes meningitis (4) and myelitis (1).

* Percentages of cases with each type were compared with Chi-square test or Fisher’s exact test (where 20% cells have expected count less than 5).

n.s: adjusted *p*≥0.05, not significant

Gastrointestinal disorders occurred in 1024 (51%) patients and more frequently in acute (53%) and subacute (48%) cases compared to chronic cases (33%) (adjusted *p*<0.05). Haematological involvement occurred in 696 (34%) patients and more commonly in the acute group (36%) than in the subacute group (28%) (adjusted *p* = 0.01). A total of 24% of male patients had epididymo-orchitis and 6% of female patients suffered from Pelvic Inflammatory Disease (PID). Respiratory involvement was detected in 122 cases (6%), the majority of which (92%) were in acute cases. Most cases of respiratory involvement presented as bronchitis (75%). Cardiovascular involvement occurred in 13 cases (0.6%). Of these, 2 had endocarditis and 11 had pericarditis. Central nervous system (CNS) involvement occurred in 5 cases (0.3%), including meningitis (4 cases) and myelitis (1 case). A total of 14 cases (0.7%) manifested as cutaneous complaints and 1 case was diagnosed as uveitis.

### Follow up and outcomes

A total of 1321 cases (65% of total cases) responded to our telephone interview more than 450 days after discharge from hospital. This was long enough to observe treatment outcome (S2 Table). Of these, 812 cases (61.5%) fully recovered, 499 cases (37.7%) were unresolved and 10 cases (0.8%) died. Unresolved cases were defined as patients with continued symptoms after discharge. In fatal cases, a 56-year-old male farmer died from endocarditis caused by Brucella after about 60 days of illness onset, while other patients died from other diseases. Of the 499 unresolved cases, there were 74 cases (14.8%) still under medical care at the time of follow-up.

### Risk factors associated with outcomes

#### Univariate analysis

In the univariate logistic regression model (Table 5), factors associated with unfavourable outcome of brucellosis were: age ≥45 years (OR = 1.78, 95% CI 1.42–2.23, *p<*0.01); arthralgia (OR = 1.45, 95% CI 1.15–1.83, *p =* 0.002); back pain (OR = 1.58, 95% CI 1.25–2.00, *p<*0.01) and joints tenderness (OR = 2.09, 95% CI 1.42–3.08, *p<*0.01). The risk of poor prognosis increased as illness duration increased. However, cases with fever (OR = 0.77, 95% CI 0.61–0.98, *p =* 0.030), headache (OR = 0.73, 95% CI 0.55–0.95, *p =* 0.021), haematologic involvement (OR = 0.67, 95% CI 0.52–0.86, *p =* 0.002), aminotransferase elevation (OR = 0.74, 95% CI 0.59–0.93, *p =* 0.011), culture positive (OR = 0.67, 95% CI 0.53–0.85, *p =* 0.008) or treatment with doxycycline combined with rifampicin, were more likely to fully recover.

**Table 5:** Univariate logistic regression analysis of risk factors for unfavourable prognosis of brucellosis cases.

Variables	Recovered cases N = 812	Unresolved cases N = 481	OR	95% CI	*p* *
Demographic features					
Male	648 (80)	365 (76)	0.81	0.62–1.06	0.119
Age≥45	333(41)	265 (55)	1.78	1.42–2.23	<0.0001
Duration of illness (days)					
≤7	139 (17)	53 (11)	Reference		
~30	251 (31)	124 (25)	1.33	0.91–1.96	0.14
~90	294 (36)	189 (38)	1.70	1.17–2.45	0.005
~180	80 (10)	67 (13)	2.20	1.40–3.48	0.0007
≥180	48 (6)	66 (13)	3.40	2.08–5.55	<0.0001
Symptoms					
Fatigue	550 (68)	316 (66)	0.87	0.68–1.10	0.240
Arthralgia	470 (58)	317 (66)	1.45	1.15–1.83	0.002
Sweating	442 (54)	273 (57)	1.07	0.86–1.35	0.545
Back pain	259 (32)	216 (45)	1.58	1.25–2.00	0.0001
Lack of appetite	209 (26)	121 (25)	0.94	0.72–1.21	0.611
Headache	209 (26)	99 (21)	0.73	0.55–0.95	0.021
Chills	128 (16)	65 (14)	0.82	0.59–1.12	0.213
Myalgia	101 (12)	66 (14)	1.07	0.76–1.49	0.703
Sleep disturbance	87 (11)	56 (12)	1.05	0.74–1.51	0.775
Cough	77 (9)	46 (10)	0.91	0.62–1.34	0.626
Signs					
Fever†	542 (67)	296 (62)	0.77	0.61–0.98	0.030
Weight loss	169 (21)	90 (19)	0.87	0.66–1.16	0.867
Joints tenderness	53 (7)	63 (13)	2.09	1.42–3.08	0.0002
Testis swelling	30 (4)	13 (3)	0.81	0.43–1.56	0.534
Splenomegaly	517 (63)	350 (73)	1.46	1.14–1.86	0.002
Hepatomegaly	209 (26)	101 (21)	0.76	0.63–1.08	0.154
Laboratory findings					
Haematologic involvement	265 (33)	120 (25)	0.67	0.52–0.86	0.002
CRP >10 mg/L	332 (41)	188 (39)	0.88	0.69–1.12	0.289
ESR elevation	567 (70)	332 (69)	0.92	0.69–1.21	0.531
Aminotransferase elevation	352 (43)	173 (36)	0.74	0.59–0.93	0.011
Culture positive	402 (52)	197 (42)	0.67	0.53–0.85	0.008
SAT ≥400	518 (64)	322 (65)	1.05	0.83–1.33	0.676
Treatment					
Doxycycline+Rifampicin	420 (52)	228 (47)	0.71	0.52–0.95	0.022
Doxycycline+Rifampicin+ Levofloxacin	244 (30)	143 (30)	0.79	0.54–1.03	0.076
Other regimens	148 (18)	110 (23)	Reference		

Data are no. (%) of cases, unless otherwise indicated. Percentages may not total 100 because of rounding. Abbreviation: ALT, alanine aminotransferase; AST, aspartate aminotransferase; ESR, erythrocyte sedimentation rate; CRP, C-reactive protein; Fever was defined as axillary and rectal temperature of >37.3°Cand >38.3°C, respectively.

*Medians were compared between recovered cases and unresolved cases with the Wilcoxon rank sum test. For categorical variables, percentages of cases in each category were compared with a Chi-square test or Fisher’s exact test (where 20% cells have expected count less than 5).

#### Multivariate analysis

Multivariate logistic regression analysis was used to identify factors that were independently associated with poor prognosis. Variables with *p<*0.05 in univariate analysis were included in multivariate analysis (Table 6). We found that fever, headache, arthralgia, aminotransferase elevation, culture positive and initial treatment were no longer significantly (*p*>0.05) associated with unfavourable prognosis in multivariate analysis.

**Table 6:** Risk factors for unfavourable prognosis of brucellosis cases, identified by multivariate logistic regression analysis.

Variable	OR	95% CI	*p*
Age≥45 years	1.75	1.36–2.24	<0.0001
Male	0.88	0.66–1.17	0.374
Duration of illness (days)			
≤7	Reference		
~30	1.43	0.94–2.18	0.095
~90	1.65	1.10–2.46	0.015
~180	1.75	1.06–2.88	0.029
≥180	2.99	1.75–5.10	<0.0001
Fever	0.90	0.69–1.17	0.431
Headache	0.76	0.56–1.01	0.059
Arthralgia	1.24	0.95–1.61	0.117
Back pain	1.50	1.16–1.94	0.002
Joints tenderness	1.73	1.13–2.65	0.011
Haematologic involvement	0.60	0.45–0.79	0.0003
Aminotransferase elevation	1.01	0.78–1.32	0.920
Culture positive	0.81	0.63–1.05	0.111
Initial treatment			
Doxycycline+Rifampicin	0.88	0.64–1.22	0.450
Doxycycline+Rifampicin+Levofloxacin	0.84	0.59–1.19	0.332
Other regimens	Reference		

OR odds ratio, CI confidence interval, statistic significant results are bold (p≤0.05).

We observed that age>45 years (OR = 1.75, 95% CI 1.36–2.24, *p<*0.001), back pain (OR = 1.50, 95% CI 1.16–1.94, *p =* 0.002) and joint tenderness (OR = 1.73, 95% CI 1.13–2.65, *p =* 0.011) were strongly predictive of poor outcome. Compared with cases who were treated within one week after onset, patients treated more than one week but less than one month after onset were not significantly distinguishable in prognosis. Patients who received treatment for longer than one month after onset were more likely to receive a poor prognosis: 1–3 months (OR = 1.65, 95% CI 1.10–2.46, *p =* 0.015), 3–6 months (OR = 1.75, 95% CI 1.06–2.88, *p =* 0.029), longer than 6 months (OR = 2.99, 95% CI 1.75–5.10, *p<*0.001). Brucellosis cases with abnormal haematologic findings at admission had a better clinical outcome (OR = 0.60, 95% CI 0.45–0.79, *p<*0.001).

## Discussion

This study retrospectively collected data from the medical records of 2041 patients with laboratory confirmed brucellosis and followed up their clinical outcomes. The clinical features of human brucellosis were described and risk factors for unfavorable prognosis were assessed systematically. The results from this study provide a theoretical basis for clinical diagnosis, treatment and case management of human brucellosis.

The gender and age distributions were consistent with previous studies [5, 13]. A total of 87% of children under 15 years of age presented as acute cases, which suggests that children seek medical advice earlier than adults, or they have more symptoms with infection than adults and are therefore more likely to present to hospital earlier. Or children may be less likely to have localized infections, which may not present as rapidly. The primary transmission route of brucellosis in our study was through occupational exposure (97%), which is in accord with the results of epidemiologic investigations in China [3, 15]. In other endemic countries, infections occur mostly due to ingestion of unpasteurized dairy products [16, 17].

In our study, the most common clinical manifestations of human brucellosis were fatigue (67%), fever (64%), arthralgia (63%) and sweating (54%), of which the rate was lower than that reported by the WHO [18]. We found that patients in different clinical stages presented with different clinical features. Systematic manifestation, such as fever, lack of appetite and weight loss, were more frequent in acute cases, while arthralgia, back pain and joint tenderness occurred more frequently in patients with longer disease durations. Similar findings have been described in a study done in Turkey [19].

Elevated liver enzymes, increased CRP and increased ESR were the most common laboratory findings seen in our series, especially in acute cases (see *Table 3*). Buzgan et al. reported similar results [19]. Along with increased illness duration, we found that both the positive rates of SAT and blood culture fell. This suggests that we should use tests with higher sensitivity to diagnose subacute and chronic cases to avoid missed diagnosis. ELISA, PCR, Coombs test and bone marrow culture may be good choices for subacute and chronic patients, especially for those who have used antibiotics [20, 21].

In our study, osteoarticular involvement was observed in 64% of acute cases, 77% of subacute cases and 87% of chronic cases. This is higher than that reported by Buzgan et al. [19]and Hasanjani et al.[22]. A study done in Iran showed that sacroilitis (75.7%) was more common than spondylitis (21.4%) and peripheral arthritis (8.6%) [23], while a survey done in Turkey found that peripheral arthritis (56.5%) occurred more frequently than sacroilitis (24.6%) and spondylitis (12.3%)[19]. Our study indicated that peripheral joints (57%) were more likely to be affected by Brucella than spine (20%) and sacroiliac joints (2%).

A total of 122 patients (6%) showed evidence of respiratory involvement, which mainly manifested as acute infection (92%) with no chronic cases. This is consistent with another report done in Turkey [23]. This suggests that respiratory involvement mainly occurred in acute cases. However, the 75% of cases with respiratory abnormalities presented as bronchitis in our series, while pneumonia (68.4%) and pleural effusion (30.8%) were more commonly found in Turkey [24].

In some studies from Iran, the recovery rate of human brucellosis has been reported between 80.6% [25] and 89.49% [26], which is higher than that found in our study (61.5%). In the Sixth People's Hospital, many referral patients were received. Therefore, a more severe disease population with a greater risk of poor prognosis may have been included in the current study. A small amount of research has been done on the factors that influence prognosis of human brucellosis. In our study, we identified five independent factors that influenced whether a patient with human brucellosis received a poor prognosis: age ≥45 years, back pain, joint tenderness, long duration of illness and haematologic involvement.

Brucellosis in older patients increases the risk of poor prognosis because these patients often have some immune system dysfunction [27–29]. Other studies, however, did not find differences in outcomes according to patient age [26, 30]. In our series, patients aged ≥45 years were more likely to have unfavorable prognosis. We also found that back pain and joint tenderness were associated with increased risk of poor prognosis, which is consistent with Wu’s finding [31].

Moreover, patients who had brucellosis over a longer duration were more likely to have unfavorable prognosis after treatment. Indeed, Alavi and colleagues reported that the majority of patients who relapse had a prolonged duration of time between the appearance of symptoms and initiation of treatment [32]. Although abnormal haematologic findings have previously been shown to be a marker of poor prognosis [30, 33], we present the opposite result (OR = 0.60, 95% CI 0.45–0.79, *p =* 0.003). This may be because those who had abnormal haematologic findings were more likely to present as acute cases in our series, and acute cases tend to have a lower rate of poor outcomes. Therefore, this association could be spurious. More research is needed in this area.

Previous meta-analyses have reached different conclusions regarding the preferred regimens for brucellosis [34, 35]. Generally, dual or triple regimens are advisable. In our series, multivariate analyses showed no significant differences between each combination therapy. However, since the data on real treatment duration and patient compliance after discharge were insufficient, this conclusion about the association between regimens and treatment outcome is substantially underpowered. This would need to be further investigated in a prospective study.

Our study was limited to available data of brucellosis cases identified through retrospective analysis. Cases included in this study were all from a provincial tertiary hospital, where many severe patients were referred. This might explain why the recovery rate observed in our series is lower than that identified in previous studies. Clinical management was uncontrolled, pathological data and medication compliance data were not available, and relapse and reinfection were hard to distinguish from treatment failure. Any differences in outcome cannot be interpreted to be due to the use of antibiotics since there are no clear diagnostic criteria for measuring real cure, and telephone follow-up may decrease the validity of the findings.

In summary, brucellosis cases have a high rate of chronicity. The longer the illness duration, the more difficult brucellosis is to cure. To improve the clinical management of brucellosis in China, early diagnosis and treatment should be of high priority. Age ≥45 years, back pain, and joint tenderness are risk factors of adverse prognosis of brucellosis. Longer courses of treatment should be considered for older patients and those with osteoarticular involvement. The positive rate of blood culture and SAT may gradually decrease with the progress of the condition. Therefore, sensitive diagnostic methods should be used to identify subacute and chronic brucellosis cases to avoid missed diagnosis. Brucellosis patients may show different characteristics in different clinical stages: abnormal laboratory findings and respiratory involvement may be more frequent in acute cases, osteoarticular involvement may be more frequent in subacute and chronic cases.

## Supporting information

S1 Table Definition of focal involvements. (DOCX)

S2 Table Outcome of 1321 respondents by telephone interview, Xinjiang, China, March-April 2016. (DOCX)

S3 Table Change of laboratory findings of 293 acute brucellosis cases after discharge, Xinjiang, China, 2014. (DOCX)

S1 Fig Laboratory findings of 2041 brucellosis cases during clinical stage, Xinjiang, China, 2014. (TIFF)

S2 Fig Percentage of patients positive by standard tube agglutination test (SAT) by days from onset, only acute and subacute cases included based on outpatient follow-up data. (TIFF)

S3 Fig Ethical approval form-original. (TIF)

S1 Text Ethical approval form-English version. (DOCX)

S2 Text Investigation questionaire- English version. (DOCX)

S3 Text Investigation questionaire- Chinese version. (DOCX)

S1 Checklist Clinical studies checklist. (DOCX)
