# Correction: Clinical features of 2041 human brucellosis cases in China

**DOI:** 10.1371/journal.pone.0211102

**Published:** 2019-01-16

**Authors:** The PLOS ONE Staff

There is an error in affiliation 4 and 5 for authors Shengjie Lai and Hongjie Yu. Affiliation 4 should be: WorldPop, Department of Geography and Environment, University of Southampton, Southampton, UK. Affiliation 5 should be: School of Public Health, Fudan University, Key Laboratory of Public Health Safety, Ministry of Education, Shanghai, China. The publisher apologizes for the error[s].
