# Correction: Correction: Clinical features of 2041 human brucellosis cases in China

**DOI:** 10.1371/journal.pone.0213558

**Published:** 2019-03-04

**Authors:** The PLOS ONE Staff

The affiliation for the twelfth author is incorrect. Hongjie Yu is not affiliated with #4 but with #1 and #5:

Division of Infectious Diseases, Key Laboratory of Surveillance and Early–warning on Infectious Disease, Chinese Center for Disease Control and Prevention, Beijing, China.

School of Public Health, Fudan University, Key Laboratory of Public Health Safety, Ministry of Education, Shanghai, China. The publisher apologize for the error.
