# Correction: Clinical features of 2041 human brucellosis cases in China

**DOI:** 10.1371/journal.pone.0219110

**Published:** 2019-06-25

**Authors:** Yujing Shi, Hui Gao, Georgios Pappas, Qiulan Chen, Mei Li, Jun Xu, Shengjie Lai, Qiaohong Liao, Wenwen Yang, Zhongtao Yi, Zulaguli Rouzi, Hongjie Yu

There is an error in affiliation 3 for author Georgios Pappas. The correct affiliation 3 is: Institute of Continuing Medical Education of Ioannina, Greece.
