# Supplementary material for: Clinical features of 2041 human brucellosis cases in China
Source: PLoS One. 2018 Nov 26;13(11):e0205500. doi: 10.1371/journal.pone.0205500 (PMC6258468; doi:10.1371/journal.pone.0205500)
Supplement: S1 Table — (DOCX) [file pone.0205500.s001.docx]

# S1 Table. Definition of focal involvement

| **Complication/relapse** | **Definition** |
| --- | --- |
| ***Osteoarticular involvement*** | Diagnosis of osteoarticular system complications was determined by the finding of swelling, effusion and limitation of motion in an involved joint and was confirmed by radiological signs (MRI, CT, X-ray), evaluated independently by both clinician and radiologist |
| ***Central nervous system involvement*** | The diagnosis of central nervous system involvement was based on symptoms or clinical findings of neurologic syndrome presenting during the course of conformed cases that not explained by any other neurological disease. |
| ***Cardiovascular involvement*** | Cardiovascular involvement was diagnosed by cardiac murmur, retrosternal pain and was confirmed by the abnormal findings of ECG or UCG. |
| ***Hematologic involvement*** | Hematologic involvement was defined as hematologic abnormalities in laboratory and clinical findings (signs of anemia or hemorrhage), excluding other possible causes. Anemia was defined as hemoglobin level of <130 g/L in male, <120g/L in female and children; thrombocytopenia was defined as platelet count of <100×109/L; leukopenia was defined as leukocyte count of <4×109/L, and leukocytosis was defined as leukocyte count of ＞10×109/L。 |
| ***Gastrointestinal involvement*** | Gastrointestinal complications were considered if there were any signs or symptoms of involvement such as nausea/vomiting, diarrhea, constipation, or abdominal tenderness. Hepatomegaly and splenomegaly were diagnosed by abdominal B-ultrasound. Abnormal liver function was defined as alanine transaminase elevation(ALT >40 U/L or AST >42 U/L) and/or bilirubin elevation (total bilirubin >18.6 umol /L) without any other etiological explanation. |
| ***Genitourinary involvement*** | The diagnosis of orchitis, epididymitis and pelvic inflammatory disease was based on symptoms and inflammatory signs(such as orchialgia and, testis swelling in males and low back pain, lower abdominal pain in females) and was conformed by the abnormal findings of ultrasonography. |
| ***Respiratory involvement*** | Respiratory complications was defined as the presence of bronchitis, pneumonia or pleural adhesions, which mainly manifested as cough, expectoration, abnormal breath sounds and abnormal findings of chest X-ray, CT or MRI, excluding other possible causes. |
| ***Cutaneous involvement*** | Cutaneous complications was defined as the presence of rashes, purpura and petechiaes, erythema nodosum, ulcers or abscess during the course of conformed cases that not explained by any other disease. |
| ***Ocular involvement*** | The diagnosis of uveitis was based on symptoms and signs of eyes (such as red and swollen eyes, watery eyes or blurred vision)or abnormal ophthalmologic examination. |
