# Supplementary material for: Clinical features of 2041 human brucellosis cases in China
Source: PLoS One. 2018 Nov 26;13(11):e0205500. doi: 10.1371/journal.pone.0205500 (PMC6258468; doi:10.1371/journal.pone.0205500)
Supplement: S2 Table — (DOCX) [file pone.0205500.s002.docx]

# S2 Table. Outcome of 1321 respondents by telephone interview, Xinjiang, China, March-April 2016

| Outcome | Acute (N=1001) | Subacute (N=227) | Chronic (N=43) | Total  (N=1321) |
| --- | --- | --- | --- | --- |
| Recovered | 653 (65.2) | 137 (49.5) | 22 (51.2) | 812 (61.5) |
| Unresolved | 342 (34.2) | 137 (49.5) | 20 (46.5) | 499 (37.7) |
| Death* | 6 (0.6) | 3 (1.0) ^*^ | 1 (2.3) | 10 (0.8) |

There are 720 cases (519 in acute, 169 in subacute and 32 in chronic) lost to follow up.

* Only one case died from endocarditis caused by Brucella, and other death cases died from other diseases.
