# Supplementary material for: Clinical features of 2041 human brucellosis cases in China
Source: PLoS One. 2018 Nov 26;13(11):e0205500. doi: 10.1371/journal.pone.0205500 (PMC6258468; doi:10.1371/journal.pone.0205500)
Supplement: S3 Table — (DOCX) [file pone.0205500.s003.docx]

# S3 Table. Change of laboratory findings of 293 acute brucellosis cases after discharge, Xinjiang, China, 2014

| Variables | | At hospital admission | Follow-up after discharge | *p** |
| --- | --- | --- | --- | --- |
|  |  | 0-2 months from onset  (n=293) | 6-12 months from onset  (n=293) |  |
| Haematology | |  |  |  |
| Anemia† | | 66(23) | 6(2) | <0.0001 |
| Median Hb (IQR) | | 127 (117-139) | 148 (136-158) | <0.0001 |
| Leukopenia, <4×10^9^/L | | 28 (10) | 18 (6) | 0.12 |
| Leukocytosis, >10×10^9^/L | | 22 (8) | 16 (5) | 0.31 |
| Median WBC count | | 5.9 (4.9-7.4) | 6.3 (5.2-7.5) | 0.14 |
| Lymphopenia, <0.8×10^9^/L | | 0 | 0 | - |
| Lymphocytosis, >4×10^9^/L | | 64 (22) | 78 (27) | 0.18 |
| Median LYM count(IQR) | | 3.0 (2.2-3.9) | 3.1 (2.5-4.1) | 0.03 |
| Thrombocytopenia,<100×109/L | | 6 (2) | 5(2) | 0.76 |
| Median PLT count(IQR) | | 222 (178-278) | 213 (183-252) | 0.08 |
| Serum biochemistry | |  |  |  |
| ALT>40 U/L | | 134 (46) | 27 (9) | <0.0001 |
| Median ALT (IQR) | | 38 (24-59) | 20 (14-27) | <0.0001 |
| AST>42 U/L | | 75 (27) | 5(2) | <0.0001 |
| Median AST (IQR) | | 29 (21-43) | 20 (16-23) | <0.0001 |
| Bilirubin >18.6 umol /L | | 44 (15) | 31 (11) | 0.11 |
| Median bilirubin (IQR) | | 10.7 (15) | 10.2 (7.6-14.2) | 0.39 |
| Urea nitrogen >7.14 mmol/L | | 9 (3) | 17 (6) | 0.09 |
| Median urea nitrogen (IQR) | 4.1 (3.4-5.0) | | 4.9 (4.0-5.9) | <0.0001 |
| Creatinine >124 umol/L | 3 (1) | | 1 (0.4) | 0.32 |
| Median creatinine (IQR) | 60 (52-68) | | 64 (55-72) | 0.005 |
| SAT ≥200 | 277 (95) | | 90 (31) | <0.0001 |
| Median SAT titer(IQR) | 400 (200-400) | | 50 (50-200) | <0.0001 |

Data are no. (%) of cases, unless otherwise indicated. Percentages may not total 100 because of rounding.

Abbreviation: IQR, inter quartile range; ALT, alanine aminotransferase; AST, aspartate aminotransferase; STA, standard tube agglutination test;

† Anemia: female and children >110 g/L , male>120 g/ L.

*Medians were compared between each group with the Wilcoxon rank sum test. For categorical variables, percentages of cases in each group were compared with Chi-square test or Fisher’s exact test (where 20% cells have expected count less than 5).
