# Supplementary figures and images for: Clinical features of 2041 human brucellosis cases in China
Source: PLoS One. 2018 Nov 26;13(11):e0205500. doi: 10.1371/journal.pone.0205500 (PMC6258468; doi:10.1371/journal.pone.0205500)

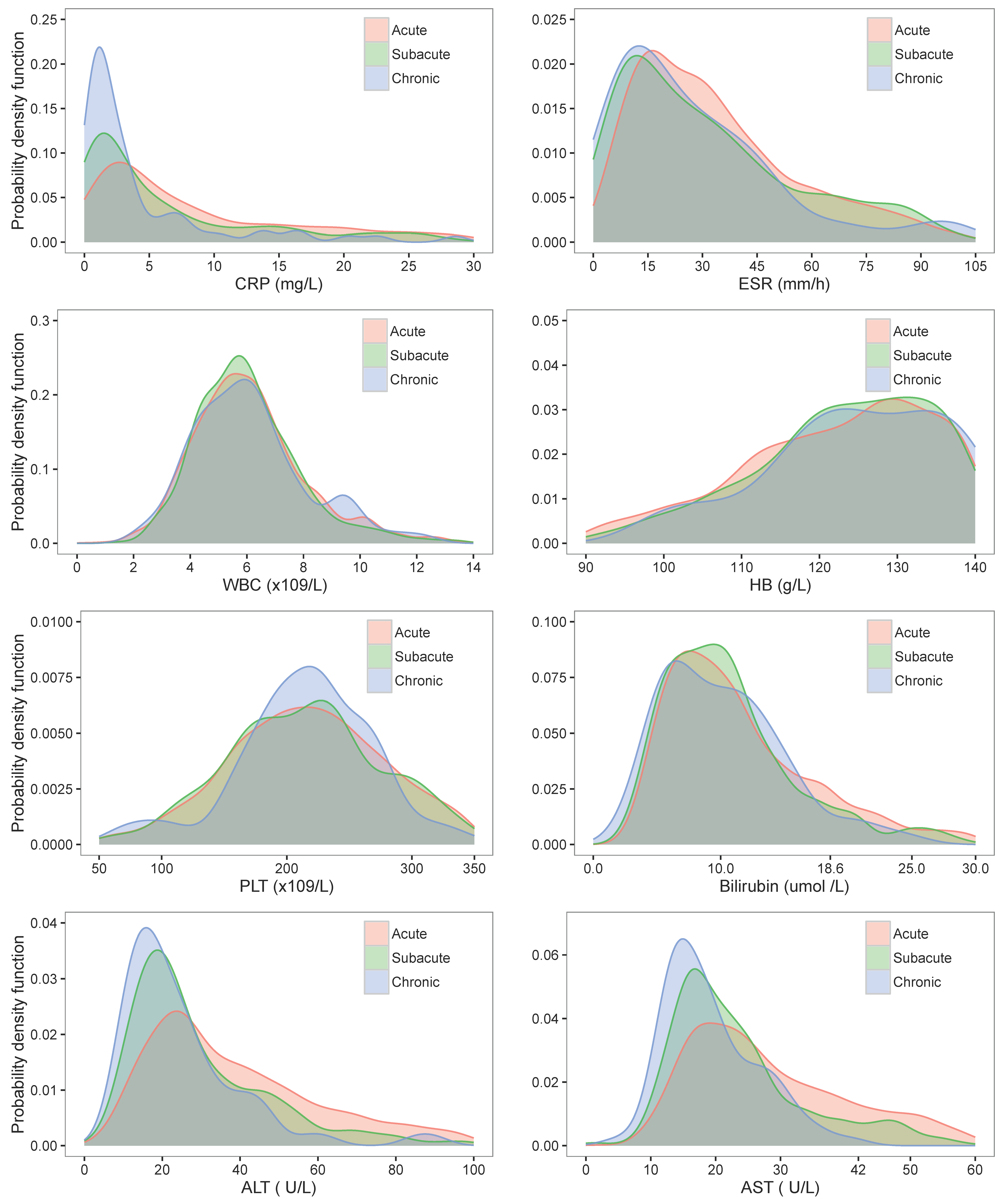

Supplement: S1 Fig — (TIFF) [file pone.0205500.s004.tiff]

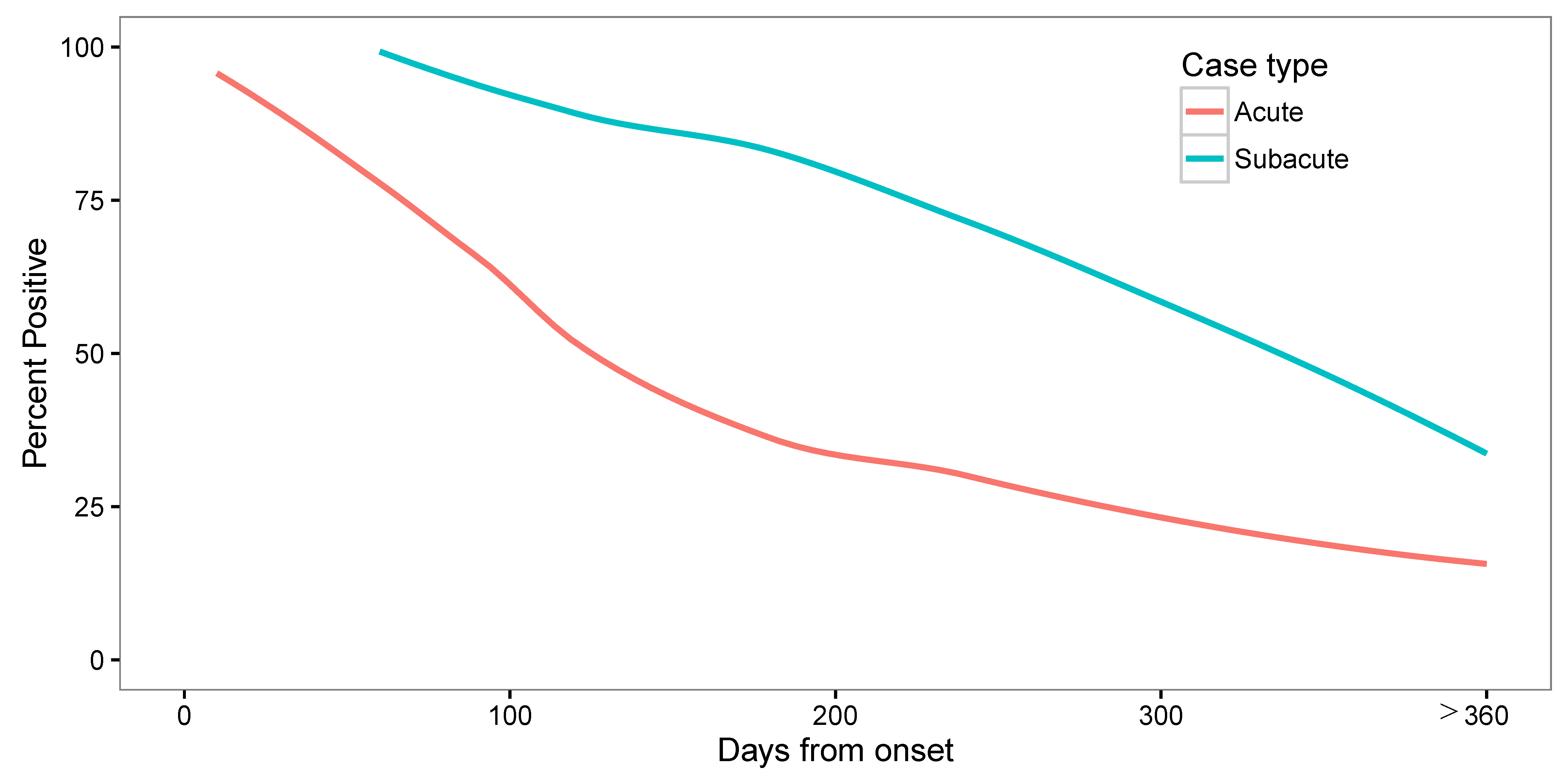

Supplement: S2 Fig — (TIFF) [file pone.0205500.s005.tiff]

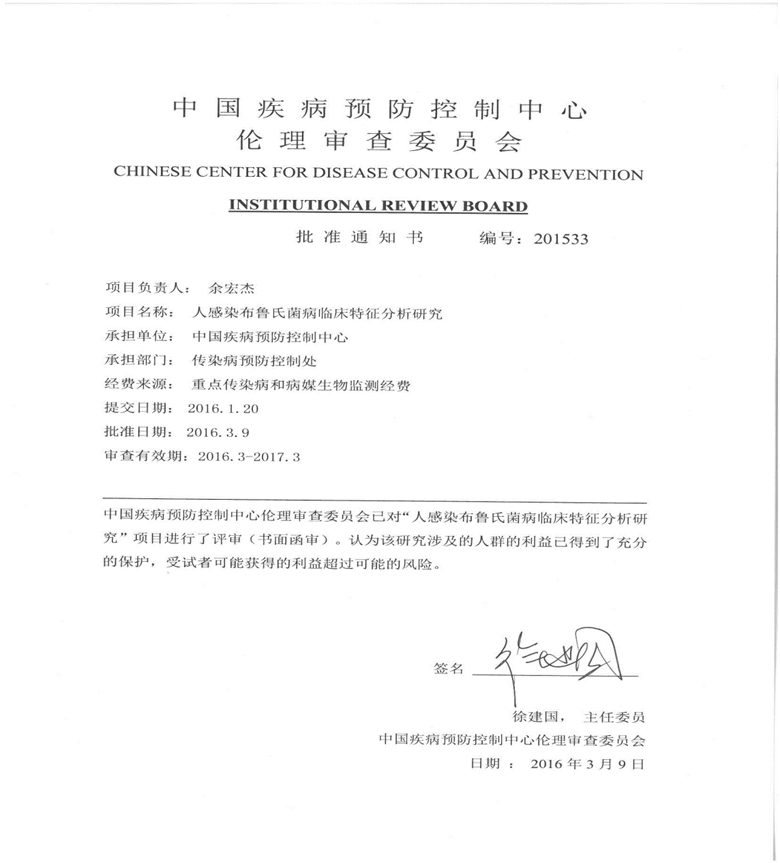

Supplement: S3 Fig — (TIF) [file pone.0205500.s006.tif]
