# Supplementary material for: Clinical features of 2041 human brucellosis cases in China
Source: PLoS One. 2018 Nov 26;13(11):e0205500. doi: 10.1371/journal.pone.0205500 (PMC6258468; doi:10.1371/journal.pone.0205500)
Supplement: S1 Text — (DOCX) [file pone.0205500.s007.docx]

CHINESE CENTER FOR DISEASE CONTROL AND PREVENTION

**INSTITUTIONAL REVIEW BOARD**

NO. 201533

[Principal](http://cn.bing.com/dict/search?q=Principal&FORM=BDVSP6&mkt=zh-cn) [Investigator](http://cn.bing.com/dict/search?q=Investigator&FORM=BDVSP6&mkt=zh-cn): Hongjie Yu

Project Name: Analysis of Clinical Characteristic of human Brucellosis

Implementing Unit: China Center for Disease Control and Prevention

Implementing Department: Division of Infectious Diseases

Sources of Funding: Surveillance funds of key infectious diseases and vector-borne diseases

Submission Date: 2016.1.20

Approval Date: 2016.3.9

Term of Validity: 2016.3-2017.3

The project of Analysis of Clinical Characteristic of human Brucellosis has been reviewed by Chinese Center for Disease Control and Prevention Institutional Review Board. We consider that the benefits of participants involved in this research have been fully protected and outweigh the risks.

Authorized by: Jianguo Xu

Chairman of Chinese Center for Disease Control and Prevention Institutional Review Board

Date: 2016-3-9
