# Supplementary material for: Clinical features of 2041 human brucellosis cases in China
Source: PLoS One. 2018 Nov 26;13(11):e0205500. doi: 10.1371/journal.pone.0205500 (PMC6258468; doi:10.1371/journal.pone.0205500)
Supplement: S2 Text — (DOCX) [file pone.0205500.s008.docx]

**Analysis of Clinical Characteristic of human Brucellosis**

**Questionnaire Survey**

**CODE** □□□□□

**Part I Basic Information of the Case**

1. Patient No.：
2. Sex： □ M □ F
3. If the patient is female, whether she is pregnant? □ Y □ N □ ?
4. Age：
5. Ethnic Group：
6. Occupation：（1）Kindergarten children（2）Scattered children（3）Student（4）Teacher（5）Nanny（6）Practitioner of catering trade（7）Business service（8）Medical personnel（9）Worker（10）Migrant worker（11）Farmer（12）Herdsman（13）Fisherman（14）Civil servants（15）Retired people（16）Job seeker（17）Other, please specify:
7. Residence： □ Urban □ Rural

**Part II Past Medical History ＆ Family Medical History**

1. Days from onset to admission: days
2. Case type: □ Acute (<2 months) □ Subacute (2-12 months) □ Chronic (>12 months)
3. Any health seeking behavior before hospitalization after onset?

□ Y □ N □ ?

1. Diagnosed as brucellosis when initially visiting the doctor?

□ Y □ N □ ?

14a if no, then diagnosed as ?

1. Has anyone of patient’s family members suffered from brucellosis during last 2 years?

□ Y □ N □ ?

**Part III Exposure History**

1. Did you have any exposure history before the onset of brucellosis?

□ Y □ N □ ?


14a. Animals you have contact with (multiple choice): □ Cow □ Sheep □ Pig

□ Dog □ Deer □ Other, please specify：

14b.The main route of exposure (multiple choice)：

□ Raising □ Slaughter □ Product (meat, milk, fur and feather)processing

□ Delivering lambs □ Transporting □Deal □ Veterinarian □Vaccination □Looking after diseased animals □Laboratory □Cleaning animal feces

□Cleaning livestock shed □Living with livestock □Vaccine production worker

□Other, please specify:

14c. Have you ever eaten undercooked meat of cattle or sheep, or have you ever drunk raw milk or dairy product?

□ Y □ N □ ?

**Part IV Symptoms and Signs**

1. Onset Date： m /d /y
2. Diagnosed Date：m /d /y
3. Do you have the following symptoms and signs ?

| **Symptom** |  | **Sign** |  |
| --- | --- | --- | --- |
| Fever | 1. Y ② N | Fever | 1. Y ② N |
| Sweating | 1. Y ② N | Rash | 1. Y ② N |
| Arthralgia | 1. Y ② N | Hepatomegaly | 1. Y ② N |
|  | 1. Single joint 2. Multi-joint |  |  |
|  | Location： |  |  |
| Limitation of motion | 1. Y ② N | Splenomegaly | 1. Y ② N |
| Chills | 1. Y ② N |  |  |
| Headache | 1. Y ② N | Lymphadenopathy | 1. Y ② N |
| Fatigue | 1. Y ② N | Jaundice | 1. Y ② N |
| Lack of appetite | 1. Y ② N | Cardiac murmur | 1. Y ② N |
| Myalgia | 1. Y ② N | CNS abnormalities | 1. Y ② N |
| Joint and back pain | 1. Y ② N | Spinal tenderness | 1. Y ② N |
| Abdominal pain | 1. Y ②N | Joints tenderness | 1. Y ② N |
|  |  |  | 1. Single joint 2. Multi-joint |
|  |  |  | Location： |
| Weight loss | 1. Y ② N | Joints swelling | ①Y ② N |
| Nausea/vomiting | 1. Y ② N |  |  |
| Constipation | 1. Y ② N | joint deformity | 1. Y ② N |
| Diarrhoea | 1. Y ② N |  |  |
| Cough | 1. Y ② N | Testis swelling | 1. Y ② N |
| Sleep disturbance | 1. Y ② N |  |  |
| Scrotal pain | 1. Y ② N | Others(please specify)： | |
| Other(please specify)： | |  |  |

*Fever* was defined as axillary and rectal temperature of ＞37.3℃ and ＞38.3℃, respectively.*

**Part V Focal involvement**

| **Focal Involvement** | **Involved** | **Specific Diagnosis** | **Special Examination** |
| --- | --- | --- | --- |
| Osteoarticular system | 1. Y ②N | 1. Sacroiliitis②Spondylitis, location: (cervical, thoracic, lumbar)   ③Peripheral arthritis：□Single joint □Multi-joint; location  ④Other： | 1. CT □N □P 2. MRI □N □P 3. X-ray □N □P 4. other： |
| CNS | 1. Y ②N | 1. [Meningitis](http://cn.bing.com/dict/search?q=meningitis&FORM=BDVSP6&mkt=zh-cn) ②Extradural abscess   ③[Cerebral](http://cn.bing.com/dict/search?q=cerebral&FORM=BDVSP6&mkt=zh-cn) [hemorrhage](http://cn.bing.com/dict/search?q=hemorrhage&FORM=BDVSP6&mkt=zh-cn) ④Peripheral neuropathy ⑤Other： | ① CSF □N □P   1. CT □N □P 2. MRI □N □P 3. Other： |
| Cardiovascular system | 1. Y ②N | ①Endocarditis ②Aneurism  ③Valvular vegetations  ④Other: | 1. UCG □N □P 2. ECG □N □P 3. Other： |
| Gastrointestinal  system | 1. Y ②N | ①Hepatomegaly ②Splenomegaly  ③Diarrhoea ④Jaundice ⑤Others: | ① Liver function test  ALT IU/L  AST IU/L  TBil umol/L   1. Ultrasound □N □P |
| Genitourinary system | 1. Y ②N | 1. Testitis ②Epididymitis ③PID   ④Appendagitis⑤Other： | 1. Kidney function test   Creatinine umol/L  BUN mmol/L  ②Ultrasound □N □P |
| Respiratory system | 1. Y ②N | ①Bronchitis ②Pneumonnia  ③Pleural ④Other： | 1. DR □N □P 2. CT □N □P   ③Sputum culture □N □P |
| Hematologic system | 1. Y ②N | 1. Anemia ② Leukopenia   ②Leukocytosis ④Lymphopenia ⑤Lymphocytosis ⑥Thrombocytopenia  ⑦Other： | RBC ×10^12/L  WBC ×10^9/L  LYM x10^9^/L；  Hb g/L, PLT ×10^9/L |
| Cutaneous complications | 1. Y ②N | ①Rash ②Petechia/purpura  ③Skin ulcer ④Other |  |
| Ocular complications | 1. Y ②N | ①Uveitis ②Keratitis  ③Conjunctivitis ④Other |  |
| Other organs or system：please specify | | | |

**Part VI Laboratory Findings**

1. Bacterial culture □ Y □ N
2. Treatment with antibiotics before taking sample：□ Y □ N
3. Specimen type：①blood ②synovial fluid ③CSF ④bone marrow ⑤Other (please specify)：
4. Sampling date：m /d /y
5. Result of bacterial culture：①Positive ②Negative ③Unknown
6. Report date: m /d /y
7. RBPT: ①Positive ②Negative
8. SAT: ①Positive ②Negative

25a. SAT titer:

25b. Sampling date：m /d /y

25c. Report date：m /d /y

1. CRP: mg/L
2. ESR: mm/h

**Part VII Treatment**

1. Start antibiotic treatment on: m /d /y

Antibiotic including:

1. Doxycycline
2. Rifampicin
3. Streptomycin
4. Sulfonamides
5. Tobramycin
6. Levofloxacin
7. Ciprofloxacin
8. Fluoroquinolones
9. Cephalosporins
10. Others，please specify

**Part VIII Hospital Discharge**

| Admission Date | m /d /y |
| --- | --- |
| Discharge Date | m /d /y |
| Complaint |  |
| Discharge Diagnosis |  |
| Symptom | ①Disappear completely; ② Improved; ③Not improved; ④ others: please specify |

**Part IX Reexamination after discharge**

1. The first reexamination after discharge

| Date | m /d /y |
| --- | --- |
| Type | 1. Outpatient ② Hospitalization |
| Bacterial culture | ①Positive ②Negative ③Unknown |
| SAT | 1. Positive ②Negative Titer： |
| Hematologic system | RBC ×10^12/L；WBC ×10^9/L；LYM x10^9^/L; Hb g/L； PLT ×10^9/L |
| Liver function test | ALT IU/L；AST IU/L；TBil umol/L |
| Kidney function test | Creatinine umol/L; BUN mmol/L |

1. The second reexamination after discharge

| Date | m /d /y |
| --- | --- |
| Type | 1. Outpatient ② Hospitalization |
| Bacterial culture | ①Positive ②Negative ③Unknown |
| SAT | 1. Positive ②Negative Titer： |
| Hematologic system | RBC ×10^12/L；WBC ×10^9/L；LYM x10^9^/L; Hb g/L； PLT ×10^9/L |
| Liver function test | ALT IU/L；AST IU/L；TBil umol/L |
| Kidney function test | Creatinine umol/L; BUN mmol/L |

1. The third reexamination after discharge

| Date | m /d /y |
| --- | --- |
| Type | 1. Outpatient ② Hospitalization |
| Bacterial culture | ①Positive ②Negative ③Unknown |
| SAT | 1. Positive ②Negative Titer： |
| Hematologic system | RBC ×10^12/L；WBC ×10^9/L；LYM x10^9^/L; Hb g/L； PLT ×10^9/L |
| Liver function test | ALT IU/L；AST IU/L；TBil umol/L |
| Kidney function test | Creatinine umol/L; BUN mmol/L |

1. The fourth reexamination after discharge

| Date | m /d /y |
| --- | --- |
| Type | 1. Outpatient ② Hospitalization |
| Bacterial culture | ①Positive ②Negative ③Unknown |
| SAT | 1. Positive ②Negative Titer： |
| Hematologic system | RBC ×10^12/L；WBC ×10^9/L；LYM x10^9^/L; Hb g/L； PLT ×10^9/L |
| Liver function test | ALT IU/L；AST IU/L；TBil umol/L |
| Kidney function test | Creatinine umol/L; BUN mmol/L |

1. The fifth reexamination after discharge

| Date | m /d /y |
| --- | --- |
| Type | 1. Outpatient ② Hospitalization |
| Bacterial culture | ①Positive ②Negative ③Unknown |
| SAT | 1. Positive ②Negative Titer： |
| Hematologic system | RBC ×10^12/L；WBC ×10^9/L；LYM x10^9^/L; Hb g/L； PLT ×10^9/L |
| Liver function test | ALT IU/L；AST IU/L；TBil umol/L |
| Kidney function test | Creatinine umol/L; BUN mmol/L |

1. The sixth reexamination after discharge

| Date | m /d /y |
| --- | --- |
| Type | 1. Outpatient ② Hospitalization |
| Bacterial culture | ①Positive ②Negative ③Unknown |
| SAT | 1. Positive ②Negative Titer： |
| Hematologic system | RBC ×10^12/L；WBC ×10^9/L；LYM x10^9^/L; Hb g/L； PLT ×10^9/L |
| Liver function test | ALT IU/L；AST IU/L；TBil umol/L |
| Kidney function test | Creatinine umol/L; BUN mmol/L |

1. The seventh reexamination after discharge

| Date | m /d /y |
| --- | --- |
| Type | 1. Outpatient ② Hospitalization |
| Bacterial culture | ①Positive ②Negative ③Unknown |
| SAT | 1. Positive ②Negative Titer： |
| Hematologic system | RBC ×10^12/L；WBC ×10^9/L；LYM x10^9^/L; Hb g/L； PLT ×10^9/L |
| Liver function test | ALT IU/L；AST IU/L；TBil umol/L |
| Kidney function test | Creatinine umol/L; BUN mmol/L |

**Part X Follow up**

| Date of follow-up | m /d /y | | |
| --- | --- | --- | --- |
| Agree to participate in the study after being informed | ① Y ② N | | |
| Still taking medicine | ① Y ② N | | |
| Reason of stopping medication | 1. Medicine side effect ②Symptoms improved ③other reasons： | Withdrawal Date： | m /y |
| Symptom | ①Disappear completely | Date | m /y |
|  | ② Improved, but still have symptoms or signs; ③Not improved | | |
| Outcome assessment | ①Cured; ② Unresolved;  ③Died from: □Brucellosis □Other reasons; ④ Lost to follow up | | |

Investigated by on m /d /y

Re-checked by on m /d /y
