# Supplementary material for: Clinical features of 2041 human brucellosis cases in China
Source: PLoS One. 2018 Nov 26;13(11):e0205500. doi: 10.1371/journal.pone.0205500 (PMC6258468; doi:10.1371/journal.pone.0205500)
Supplement: S3 Text — (DOCX) [file pone.0205500.s009.docx]

**人感染布鲁氏菌病临床特征及预后问卷调查**

编码□□□□□

**第一部分 患者基本信息**

1. 患者编号：
2. 性别： □男 □女
3. 若性别为女，是否为孕妇？ □ 是 □ 否 □不详
4. 年龄： 岁
5. 民族：
6. 职业：（1）农民（2）牧民 （3）兽医（4）学生（5）教师（6）饮食从业人员（7）医务人员（8）工人（9）干部职员（10）离退人员（11）家务待业（12）婴幼儿（13）检疫员（17）其他，请注明： ­­­­­­
7. 居住： □ 城市 □乡村

**第二部分 既往史和家族史**

1. 病程： ____ 天
2. 病程分期为：① 急性期（2个月内） ②亚急性期（2-12个月） ③慢性期（大于12个月）
3. 本次发病后，是否曾因为相关症状而存在就医行为？
4. 是 ② 否 ③不详
5. 首次就诊时，是否即怀疑（或诊断）为布病？ ① 是 ② 否 ③不详

14a.若否，则当时怀疑（或诊断）为何种疾病？

1. 与您生活在一起的家人/亲戚是否罹患过布病？@# ① 是 ② 否 ③不详

**第三部分 暴露史调查**

1. 发病前是否有暴露史？□ 是 □否 □不清楚

17a.接触过的动物是（可多选）： □ 牛 □ 羊 □ 猪 □ 狗 □ 鹿 □ 其他，请注明：

17b..主要接触方式有（可多选）： □ 饲养 □ 屠宰 □ 产品（肉、奶、皮毛等）加工 □ 接羔 □ 贩运 □ 交易 □ 兽医 □ 接种疫苗 □ 照料病畜 □实验室 □打扫羊粪 □打扫棚圈 □人畜共居 □ 疫苗生产工人 □ 其他，请概要描述：

17c**.**发病前是否食用过未煮熟的牛羊肉、奶，或食用过生奶及奶制品？

□ 是 □ 否 □ 不清楚

**第四部分 症状和体征**

1. 本次发病日期; 年 月 日
2. 本次发病初次就诊日期： 年 月 日
3. 您本次发病是否有以下症状或体征？

| **症状** |  | **体征** |  |
| --- | --- | --- | --- |
| 多汗 | 1. 是 ② 否 | 发热 | ①是 ② 否 |
| 关节疼痛 | 1. 是 ② 否 | 皮疹 | ①是 ② 否 |
|  | 1. 单关节 ②多关节 | 肝肿大 | ①是 ② 否 |
|  | 部位： | 脾肿大 | ①是 ② 否 |
| 活动受限 | ①是 ②否 |  |  |
| 寒颤 | 1. 是 ② 否 | 淋巴结肿大 | 1. 是 ② 否 |
| 畏寒 | 1. 是 ② 否 |  |  |
| 头痛头晕 | ① 是 ② 否 | 皮肤黄染 | 1. 是 ② 否 |
| 乏力 | 1. 是 ② 否 |  |  |
| 食欲不振 | 1. 是 ② 否 | 心脏杂音 | 1. 是 ② 否 |
| 肌肉疼痛 | 1. 是 ② 否 | 中枢神经系统异常 | 1. 是 ② 否 |
| 腰背痛 | 1. 是 ② 否 |  |  |
| 腹痛 | 1. 是 ②否 | 脊柱压痛 | 1. 是 ② 否 |
|  |  | 关节压痛 | 1. 是 ② 否 |
| 体重下降 | 1. 是 ② 否 |  | ①单关节 ②多关节 |
| 恶心 | 1. 是 ② 否 |  | 部位： |
| 呕吐 | 1. 是 ② 否 | 关节红肿 | ①是 ② 否 |
| 便秘 | 1. 是 ② 否 |  |  |
| 腹泻 | 1. 是 ② 否 | 关节畸形 | ①是 ② 否 |
| 咳嗽 | 1. 是 ② 否 |  |  |
| 睡眠障碍 | 1. 是 ② 否 | 阴囊肿大 | ①是 ② 否 |
| 阴囊痛 | 1. 是 ② 否 |  |  |
| 其他（请注明）： | | 其他（请注明） | |

发热^*^：腋下温度＞37.3℃或肛门温度＞38.3℃

**第五部分 并发症**

| **器官或系统** | **是否累及** | **具体诊断** | **特殊检查** |
| --- | --- | --- | --- |
| 骨关节系统 | 1. 是 ②否 | ①骶髂关节炎 ②脊柱炎 ，部位 （颈椎，胸椎，腰椎）③外周关节炎：□单关节 □多关节  部位 ④其他： | ①CT □正常 □异常  ②MRI □正常 □异常  ③X线 □正常 □异常   1. 其他： |
| 神经系统 | ① 是 ②否 | ①脑膜炎 ②硬脑膜外脓肿   1. 脑出血 ④外周神经病变   ⑤其他： | ①CSF □正常 □异常  ②CT □正常 □异常  ③MRI □正常 □异常  ④其他： |
| 心血管系统 | 1. 是 ②否 | 1. 心内膜炎 ②动脉瘤   ③瓣膜赘生物  ④其他： | ①心脏彩超 □正常 □异常  ②心电图 □正常 □异常   1. 其他： |
| 消化系统 | ①是 ②否 | 1. 肝肿大 ②脾肿大 ③腹泻   ④黄疸 ⑤恶心呕吐  ⑥其他： | 1. 肝功能检查   ALT IU/L  AST IU/L  TBil umol/L  ②B超 □正常 □异常 |
| 泌尿生殖  系统 | 1. 是 ②否 | 1. 睾丸炎 ②附睾炎 ③盆腔炎 ④附件炎 ⑤其他： | ①肾功能检查  肌酐 umol/L  尿素氮 mmol/L  ②B超 □正常 □异常 |
| 呼吸系统 | ①是 ②否 | ①支气管炎 ②肺炎  ③胸膜积液 ④其他： | ①胸片 □正常 □异常   1. CT □正常 □异常   ③痰培养 □阳 □阴 |
| 血液系统 | 1. 是 ②否 | 1. 贫血 ② 白细胞减少症 ③白细胞增多症 ④淋巴细胞减少症 ⑤淋巴细胞增多症⑥ 血小板减少症⑦其他： | RBC ×10^12/L  WBC ×10^9/L  Hb g/L, PLT ×10^9/L |
| 皮肤并发症 | ①是 ②否 | 1. 皮疹 ②瘀点或紫癜   ③皮肤溃疡 ④其他 |  |
| 眼部并发症 | 1. 是 ②否 | ①葡萄膜炎 ②角膜炎  ③结膜炎 ④其他： |  |
| 其他器官或系统：请注明 | | | |

**第六部分 实验室检查**

**细菌培养**

1. 是否开展细菌培养？□ 是 □ 否
2. 采样前是否开展抗生素治疗：①是 ②否
3. 标本类型：①全血 ②关节液 ③滑囊液 ④脑脊液 ⑤骨髓 ⑥其他，请注明：
4. 采样日期： 年 月 日
5. 细菌培养结果：①阳性 ②阴性 ③不确定
6. 结果报告日期： 年 月 日

**血清学检测**

1. 虎红平板凝集试验(RBPT): ① 阳性 ②阴性
2. 试管凝集试验(SAT）： ① 阳性 ②阴性

25a.SAT滴度: 1: ____

25b.采样日期： 年 月 日

25c.报告日期: 年 月 日

1. C 反应蛋白： mg/L；
2. 血沉： mm/h；

**第七部分 治疗方案**

1. 本次住院启动治疗的日期: 年 月 日

使用的抗生素包括：

(1)多西环素 ①是 ②否

(2)利福平 ①是 ②否

(3)链霉素 ①是 ②否

(4)磺胺类药物 ①是 ②否

(5)妥布霉素 ①是 ②否

(6)左氧氟沙星 ①是 ②否

(7)环丙沙星 ①是 ②否

(8)氟喹诺酮类 ①是 ②否

(9)头孢菌素类 ①是 ②否

(10)其他，请注明_____________________________________________

**第八部分 出院情况**

1. 入院时间: 年 月 日
2. 出院时间: 年 月 日
3. 主诉：_________________________________
4. 出院诊断:____________________________________________
5. 症状情况：① 完全消失或基本消失；②好转；③一直未见好转；④其他，请注明_________

**第九部分 复查结果**

**出院后第一次复查**

1. 时间： 年 月 日
2. 类型： ① 门诊 ②住院
3. 细菌培养结果：①阳性 ②阴性 ③不确定
4. SAT： ① 阳性 ②阴性

SAT滴度:1: ____

1. 血液系统：

红细胞： x10^12^/L；

白细胞： x10^9^/L；

淋巴细胞： x10^9^/L；

血红蛋白： g/L；

血小板： x10^9^/L；

1. 肝功能：

谷丙转氨酶: U/L；

谷草转氨酶: U/L；

总胆红素： umol/L；

1. 肾功能：

尿素氮： mmol/L；

肌酐： umol/L；

**出院后第二次复查**

1. 时间： 年 月 日
2. 类型： ① 门诊 ②住院
3. 细菌培养结果：①阳性 ②阴性 ③不确定
4. SAT： ① 阳性 ②阴性

SAT滴度:1: ____

1. 血液系统：

红细胞： x10^12^/L；

白细胞： x10^9^/L；

淋巴细胞： x10^9^/L；

血红蛋白： g/L；

血小板： x10^9^/L；

1. 肝功能：

谷丙转氨酶: U/L；

谷草转氨酶: U/L；

总胆红素： umol/L；

1. 肾功能：

尿素氮： mmol/L；

肌酐： umol/L；

**出院后第三次复查**

1. 时间： 年 月 日
2. 类型： ① 门诊 ②住院
3. 细菌培养结果：①阳性 ②阴性 ③不确定
4. SAT： ① 阳性 ②阴性

SAT滴度:1: ____

1. 血液系统：

红细胞： x10^12^/L；

白细胞： x10^9^/L；

淋巴细胞： x10^9^/L；

血红蛋白： g/L；

血小板： x10^9^/L；

1. 肝功能：

谷丙转氨酶: U/L；

谷草转氨酶: U/L；

总胆红素： umol/L；

1. 肾功能：

尿素氮： mmol/L；

肌酐： umol/L；

**出院后第四次复查**

1. 时间： 年 月 日
2. 类型： ① 门诊 ②住院
3. 细菌培养结果：①阳性 ②阴性 ③不确定
4. SAT： ① 阳性 ②阴性

SAT滴度:1: ____

1. 血液系统：

红细胞： x10^12^/L；

白细胞： x10^9^/L；

淋巴细胞： x10^9^/L；

血红蛋白： g/L；

血小板： x10^9^/L；

1. 肝功能：

谷丙转氨酶: U/L；

谷草转氨酶: U/L；

总胆红素： umol/L；

1. 肾功能：

尿素氮： mmol/L；

肌酐： umol/L；

**出院后第五次复查**

1. 时间： 年 月 日
2. 类型： ① 门诊 ②住院
3. 细菌培养结果：①阳性 ②阴性 ③不确定
4. SAT： ① 阳性 ②阴性

SAT滴度:1: ____

1. 血液系统：

红细胞： x10^12^/L；

白细胞： x10^9^/L；

淋巴细胞： x10^9^/L；

血红蛋白： g/L；

血小板： x10^9^/L；

1. 肝功能：

谷丙转氨酶: U/L；

谷草转氨酶: U/L；

总胆红素： umol/L；

1. 肾功能：

尿素氮： mmol/L；

肌酐： umol/L；

**出院后第六次复查**

1. 时间： 年 月 日
2. 类型： ① 门诊 ②住院
3. 细菌培养结果：①阳性 ②阴性 ③不确定
4. SAT： ① 阳性 ②阴性

SAT滴度:1: ____

1. 血液系统：

红细胞： x10^12^/L；

白细胞： x10^9^/L；

淋巴细胞： x10^9^/L；

血红蛋白： g/L；

血小板： x10^9^/L；

1. 肝功能：

谷丙转氨酶: U/L；

谷草转氨酶: U/L；

总胆红素： umol/L；

1. 肾功能：

尿素氮： mmol/L；

肌酐： umol/L；

**出院后第七次复查**

1. 时间： 年 月 日
2. 类型： ① 门诊 ②住院
3. 细菌培养结果：①阳性 ②阴性 ③不确定
4. SAT： ① 阳性 ②阴性

SAT滴度:1: ____

1. 血液系统：

红细胞： x10^12^/L；

白细胞： x10^9^/L；

淋巴细胞： x10^9^/L；

血红蛋白： g/L；

血小板： x10^9^/L；

1. 肝功能：

谷丙转氨酶: U/L；

谷草转氨酶: U/L；

总胆红素： umol/L；

1. 肾功能：

尿素氮： mmol/L；

肌酐： umol/L；

**第十部分 随访调查**

| 随访时间： | 年 月 日 | | |
| --- | --- | --- | --- |
| 获知情告知后，是否愿意接受调查 | ①是 ②否 | | |
| 是否仍在服药 | ①是 ②否 | | |
| 不服药原因： | ①药物副反应 ②症状好转 ③其他 | 停药时间： | 年 月 |
| 症状情况 | ①完全消失或基本消失 | 时间： | 年 月 |
|  | ② 好转，但仍有症状和体征 | 症状或体征： | |
|  | ③未见好转 |  |  |
| 疗效判断 | ①治愈；②未治愈；   1. 死亡：因布病死亡、非布病死亡；死亡时间： 年 月 ④失访 | | |

住院记录调查员： 调查日期： 年 月 日随访调查员： 调查日期： 年 月 日

复核员： 复核日期： 年 月 日
